# Small molecule absorption by PDMS in the context of drug response bioassays

**DOI:** 10.1016/j.bbrc.2016.11.062

**Published:** 2017-01-08

**Authors:** B.J. van Meer, H. de Vries, K.S.A. Firth, J. van Weerd, L.G.J. Tertoolen, H.B.J. Karperien, P. Jonkheijm, C. Denning, A.P. IJzerman, C.L. Mummery

**Affiliations:** a Dept. of Anatomy and Embryology, Leiden University Medical Centre, Einthovenweg 20, 2333 ZC, Leiden, The Netherlands; b Leiden Academic Centre for Drug Research, Leiden University, Einsteinweg 55, 2333 CC, Leiden, The Netherlands; c Dept. of Stem Cell Biology, University of Nottingham, University Park, Nottingham, NG7 2RD, UK; d LipoCoat B.V., PO Box 217, 7500 AE, Enschede, The Netherlands; e Dept. of Developmental BioEngineering, University of Twente, Driernerlolaan 5, 7522 NB, Enschede, The Netherlands; f Dept. of Molecular Nanofabrication, University of Twente, P.O. Box 217, 7500 AE, Enschede, The Netherlands; g Dept. of Applied Stem Cell Technologies, University of Twente, P.O. Box 217, 7500 AE, Enschede, The Netherlands

**Keywords:** PDMS, Absorption, Drug screening, LipoCoat Cellbinder, PDMS coating, Microfluidics

## Abstract

The polymer polydimethylsiloxane (PDMS) is widely used to build microfluidic devices compatible with cell culture. Whilst convenient in manufacture, PDMS has the disadvantage that it can absorb small molecules such as drugs. In microfluidic devices like “Organs-on-Chip”, designed to examine cell behavior and test the effects of drugs, this might impact drug bioavailability. Here we developed an assay to compare the absorption of a test set of four cardiac drugs by PDMS based on measuring the residual non-absorbed compound by High Pressure Liquid Chromatography (HPLC). We showed that absorption was variable and time dependent and not determined exclusively by hydrophobicity as claimed previously. We demonstrated that two commercially available lipophilic coatings and the presence of cells affected absorption. The use of lipophilic coatings may be useful in preventing small molecule absorption by PDMS.

## Highlights

• Binding of different compounds to PDMS varies greatly.
• Previous reported correlations of absorption and LogP values could not be repeated.
• Topological polar surface area possibly related to compound absorption.
• A lipid based coating partially obviates compound absorption.
• Presence of cultured cells affects free drug concentration, but less than substrate.

## Graphical abstract

**Figure:**
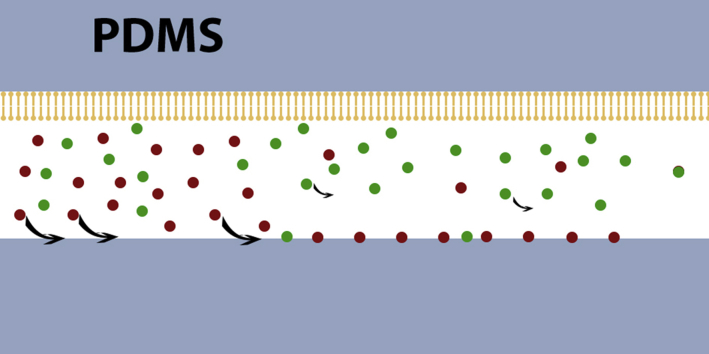

## 1 Introduction

Differences in physiology can compromise the ability of animal models to predict drug responses in humans accurately. As a result, inappropriate drugs may enter clinical trials or useful drugs discarded unnecessarily early in development. One of the most common adverse drug effects is still cardiotoxicity despite use of ion channel (“hERG”) assays to predict arrhythmic risk, illustrating the urgent need for improvement in human cardiac models [1]. In this context, the Food and Drug Association (FDA) recently initiated the Comprehensive In Vitro Proarrhythmia Assay (CIPA) (cipaproject.org), to assess cardiac drug safety using cardiomyocytes from human pluripotent stem cells. Among the models being examined are several incorporating cardiomyocytes into polymer-based devices or microtissues [2], [3], [4] rather than conventional tissue culture substrates like glass and plastic, which are orders of magnitude harder and less elastic than cells would normally encounter in tissue. Resulting stress can influence cell behavior profoundly and thus drug responses [5], [6], [7]. Soft polymer substrates like polydimethylsiloxane (PDMS) are preferred by bioengineers because they can be used to fabricate microfluidic channels, flexible membranes and provide topological cues to recapitulate blood vessels, mechanical tissue strain and induce cell orientation [8], [9], [10]. Furthermore, sensors that measure electrical activity, metabolic behavior, ion transport and force of contraction can be integrated as readouts [2], [11]. PDMS-based Organ-on-Chip devices can also be used for live cell imaging and are compatible with immunoassays, disease phenotyping and drug testing [12], [13]. Soft polymer-based microfluidic assays have long been used for multiple biological purposes [14] but PDMS most commonly because of its biocompatibility, transparency, ease of microfabrication, molding properties and tunable substrate mechanics [15]. Its elasticity has made it particularly useful in mimicking tissues that undergo cyclic stretch and strain in the body, such as the lungs or heart [2], [8], [11]. One of its drawbacks, however, is its absorption of compounds added to culture medium in bioassays, most particularly, hydrophobic small molecules like many drugs [16], [17], [18]. This reduces the available drug dose, shifts the dose response curve and thus limits the predictive value of assays.

There have been few direct comparisons of absorption of different compounds by PDMS with other (more inert) substrates and how cell culture affects absorption has not been investigated. Toepke et al. examined the absorption of hydrophobic small molecules qualitatively with a fluorescence assay but this was not quantitative [17]. Wang et al. did assess final compound concentration quantitatively over time and determined a threshold that distinguished compounds with negligible absorption from those where it was significant, based on the log P value [18], a measure of hydrophobicity. It was claimed that all compounds with a log P value greater than 2.67 would show high absorption by PDMS substrates. In addition, they tested whether glass and titanium dioxide coatings reduced compound binding. These coatings have been reported to be biocompatible [16], but the presence of cells on compound absorption or coating degradation has not been investigated. Parylene coating has also been reported [19], but it compromises important properties of PDMS, such as its elasticity and gas permeability.

Here, we determined small molecule absorption by PDMS-coated culture wells versus standard culture plastic for four drugs that affect the heart, our primary research area [20], [21] for which there is interest in knowing their toxic threshold: verapamil, bepridil, Bay K 8644 and nifedipine. Toxic thresholds can only be determined accurately if the compound availability to cells is known. We therefore investigated whether cell density affected absorption and tested the ability of two commercial coatings (available on request) to reduce absorption, without affecting biocompatibility or cell plating [22]. Finally, we combined a commercial coating with cell culture to investigate the combined effect on the free drug concentration over time of incubation.

## 2 Experimental

### 2.1 Preparation of plates

Standard tissue culture grade polystyrene (TCPS) 96-well CELLSTAR^®^ multiplates (Greiner Bio-One, Germany) were used. For PDMS absorption experiments, bottoms of wells were coated with 20 μL PDMS Sylgard 184 (Dow Corning, Auburn, MI), 1:10 wt ratio curing agent to elastomer. LipoCoat Cellbinder (LipoCoat, Enschede, The Netherlands) was used to coat oxygen plasma treated PDMS-coated 96-wells plates (PDMS Sylgard 184, Dow Corning, Auburn, MI).

### 2.2 Experimental procedure and sample retrieval

Verapamil, nifedipine (Sigma Aldrich, Saint Louis, MO), bepridil and Bay K 8644 (Tocris, United Kingdom) were diluted in dimethylsulfoxide (DMSO; Sigma Aldrich) to obtain 1 mM stock. This was diluted 1:1000 in Dulbecco's Phosphate Buffered Saline without calcium and magnesium (DPBS) or in Tyrode's buffer when cells were present, to a working concentration of 1 μM. All solutions were prepared in disposable glass vials. 250 μL of the working solution was pipetted into the TCPS or PDMS wells and incubated for 0.5, 1, 2 or 3 h before being gently mixed with a pipette and transferred to a glass vial which was sealed immediately. In addition, 250 μL of the working solution was pipetted into a glass vial for every sample group at the start of the experiment to provide baseline values (concentration at time zero). Time points were chosen to allow <10% change in absorption at the final time point for all compounds. Experiments were in duplicate. Cell culture experiments were at 37 °C; other experiments were at room temperature.

## 3 HPLC

Drug concentrations were determined by HPLC using a solvent delivery system from Gilson (Middleton, WI) equipped with a Gilson auto injector. A VI/VIZ detector (Applied Biosystems 759A; Thermo Fisher Scientific, Waltham, MA) was used, monitoring the UV absorption maximum of the compounds under investigation. A Discovery^®^ column (125 × 4.6 mm; Supelco, Bellefonte, PA) was applied, with a stationary phase of C18, 5 μM particles. As mobile phase, MeCN was used at 40–65% (depending on the compound), 5 mM SDS and 0.1% TFA. Flow speed was 0.8 ml/min and the injection volume 100 μl. Recording and processing of data was performed using ADchom software (Leiden University, the Netherlands). In every experiment a calibration curve was included to validate the system and the linearity of the compound UV absorption response. Measurements were performed in duplicate or triplicate, as indicated. Averages from individual experiments are shown in the graphs. Since replicates showed little spread, n = 2 was standard for non-biological assays.

### 3.1 Cell culture

Human Embryonic Kidney 293T (HEK293T) cells were maintained in Dulbecco's Modified Eagle Medium (DMEM) supplemented with 2 mM L-glutamine, 10 mM non-essential amino acids (NEAA), 25 U/ml penicillin, 25 μg/ml streptomycin (Thermo Fisher Scientific), and 10% fetal calf serum (FCS) (Sigma Aldrich). Culture medium was refreshed every 3–4 days. For experiments, cells were dissociated from standard 6 well tissue culture plates (Greiner Bio-One, Germany) with 0.1% trypsin and plated in 96-well plates at three densities: 9 × 10^3^, 37 × 10^3^ and 117 × 10^3^ cells per well (0.3 cm^2^) corresponding to low, medium and high surface coverage, respectively.

### 3.2 Cell staining

Cells were briefly washed with PBS before fixation with 2% neutral paraformaldehyde for 30 min at room temperature. Next, cells were permeabilized for 8 min with 0.1% Triton X-100 (Sigma Aldrich) in PBS then preincubated with 4% FCS in PBS for 1 h to block non-specific binding. Cells were incubated with PBS plus Alexa Fluor 488 Phalloidin (1:40; Invitrogen) and 4% FCS for 30 min. Finally, cells were incubated with PBS plus DAPI Nucleic Acid Stain (1:1000; Invitrogen) before glass coverslips were mounted on top of the PDMS with ProlongGold (Thermo Fisher Scientific). Between every step, cells were washed with PBS and prior to Phalloidin with PBS plus 0.05% Tween 20 (Merck, Germany).

## 4 Results

**Fig. S1:**
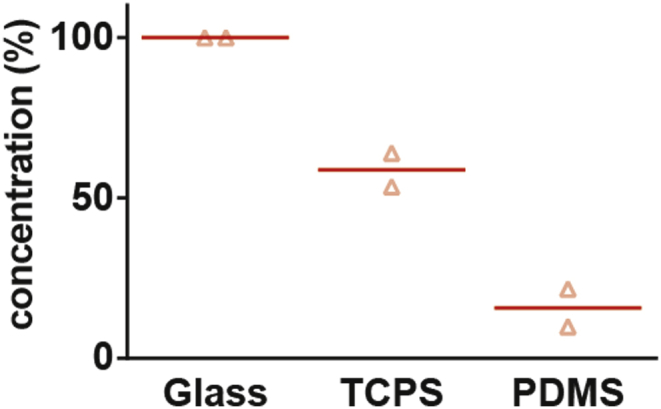
**Compound stability in glass**. Final free drug concentration of bepridil after 3 h of incubation in sealed glass containers compared to incubation in TCPS and PDMS well plates. Averages from two independent experiments are shown.

**Fig. 1:**
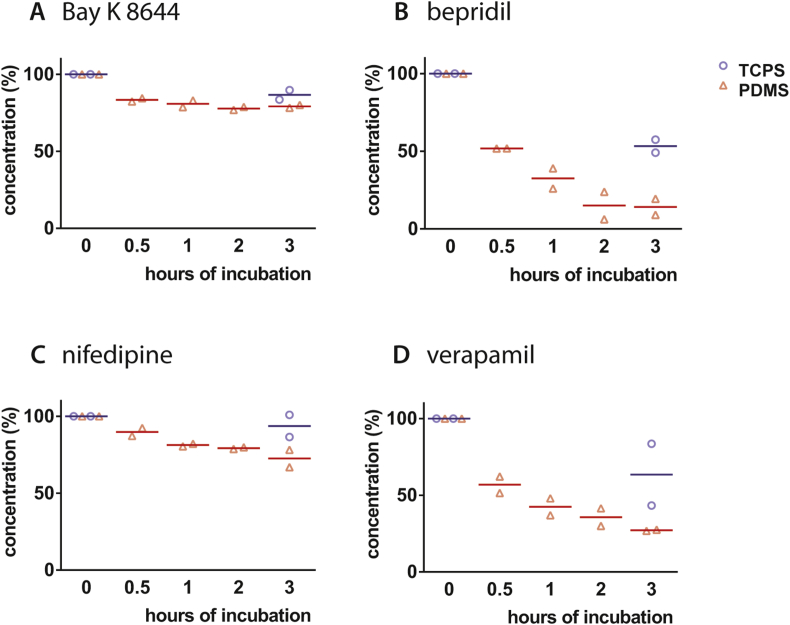
**Free drug concentration lifetime for incubation on TCPS and PDMS.** Free drug concentration after 0.5, 1, 2 and 3 h incubation on PDMS and 3 h on TCPS of A) Bay K 8644, B) bepridil, C) nifedipine and D) verapamil. Averages from two independent experiments are shown.

HPLC was used to measure the free drug concentrations in PBS. Four compounds that affect cardiac behaviour and of interest to validate in cardiotoxicity models *in vitro* were incubated for 0.5, 1, 2 and 3 h on TCPS wells with PDMS as the base and compared to the absorption by standard TCPS wells after 3 h (Fig. 1). Absorption by TCPS, measured as loss of compound from solution, was negligible or low but detectable for Bay K 8644, verapamil and nifedipine and somewhat higher for bepridil. PDMS however absorbed compounds much more variably: verapamil and nifedipine were absorbed 20–50% more than by TCPS after 3 h whilst for Bay K 8644 there was no difference between TCPS and PDMS. Bepridil was strikingly reduced more than 80% by PDMS after 3 h. To exclude compound degradation or absorption of the compounds by glass storage vials, Bay K 8644 and bepridil solutions were kept in glass containers for 3 h and compared with the starting concentrations (Fig. S1). No differences were observed. All compounds reached steady state (<10% concentration change) both on PDMS and TCPS.

**Fig. 2:**
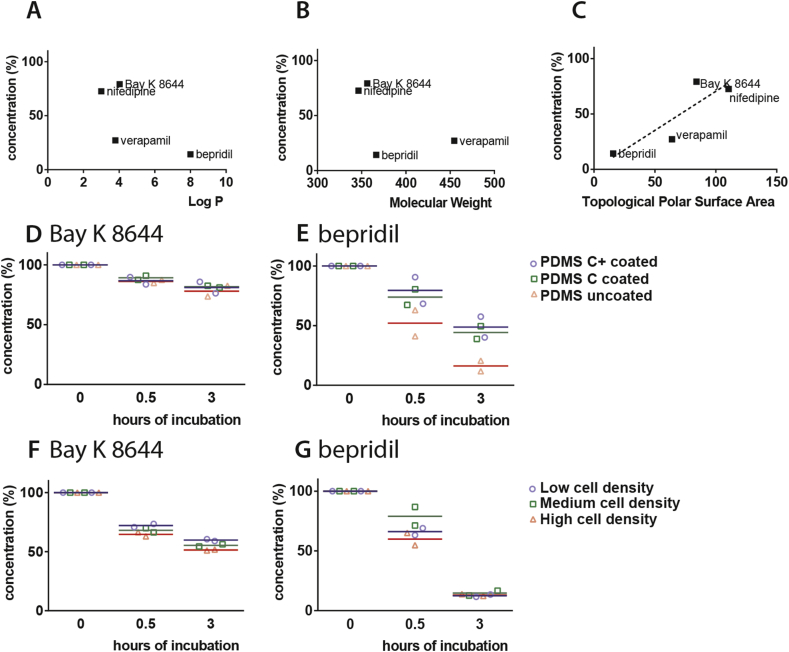
**Properties and/or conditions that influence free drug concentration lifetime on PDMS**. A) Plot of the final free drug concentration of Bay K 8644, bepridil, nifedipine and verapamil after 3 h of incubation on PDMS set out against their hydrophobicity (log P). B) Plot of the final free drug concentration of Bay K 8644, bepridil, nifedipine and verapamil after 3 h incubation on PDMS versus their molecular weight. C) Plot of the final free drug concentration of Bay K 8644, bepridil, nifedipine and verapamil after 3 h of incubation on PDMS versus their topological polar surface area and fitted with a linear best fit (dotted line, R square: 7.61). D) and E) plots of the free drug concentration of Bay K 8644 and bepridil, respectively, after 0, 0.5 and 3 h of incubation on PDMS coated with Cellbinder+, Cellbinder and without coating. F) and G) plots of the free drug concentration of Bay K 8644 and bepridil, respectively, after 0, 0.5 and 3 h of incubation at low, medium and high plating cell densities on PDMS substrates. Averages from two independent experiments are shown.

To investigate whether compound hydrophobicity, molecular weight or topological polar surface area was correlated with the absorption by PDMS, the final free drug concentration was plotted as described previously [18]. The log P values of the compounds used here were 4.03 (Bay K 8644 [23]), 8.0 (bepridil [24]) and 3.79 (verapamil [25]) and ±3 (nifedipine; provided by the manufacturer), but there was no correlation between these values and our measures of absorption (Fig. 2A). Similarly, no correlation was found between the measures of absorption and the molecular weight (Fig. 2B). The correlation between topological polar surface area and compound absorption by PDMS was linear (R square: 7.61). We also examined the effect of several mixtures of PDMS with variable elasticity on absorption [26] but all showed similar high reduction in free bepridil concentration after 3 h (data not shown) so we did not pursue this further.

Next, we coated the PDMS surface with Cellbinder, a lipid-based coating with cell binding properties [22]. To facilitate cell attachment on otherwise zwitterionic, non-fouling and cell repellent surfaces, positive surface charge had been introduced in the Cellbinder coating to promote cell adhesion. Two Cellbinder coatings were compared: Cellbinder and Cellbinder+, which has greater stability. After 3 h incubation, bepridil retained higher free drug concentration, indicating less absorption, on both coatings compared to uncoated PDMS (Fig. 2D and E). No difference was found between the two.

Apart from direct substrate effects, culture of cells could also influence free compound availability since cells may either absorb compounds or block absorption sites on the substrate. We therefore investigated whether HEK293T (kidney cell) culture density on PDMS substrates affected loss of compounds from solution. Again, low and high binders (Bay K 8644 and bepridil, respectively) were incubated at 37 °C (Fig. 2F and G). Bay K 8644 was reduced as cell density increased from 9 × 10^3^ to 37 × 10^3^ and 117 × 10^3^ cells, respectively, after 0.5 h and 3 h of incubation. A similar effect was seen for bepridil after 0.5 h although at 3 h, no differences in free concentration were evident between the three cell densities, all being equally low.

**Fig. 3:**
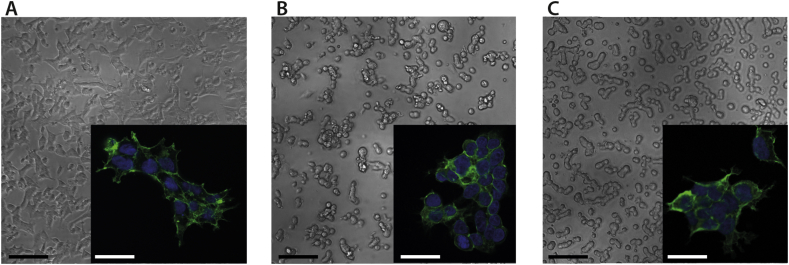
**Commercial coating for PDMS to reduce drug binding and cell plating**. Representative images of HEK293T, brightfield and fluorescent staining of the cytoskeleton (green) and nuclei (blue), on A) TCPS, B) PDMS and C) Cellbinder coated PDMS. White bars indicate 25 μm, black bars indicate 350 μm. (For interpretation of the references to colour in this figure legend, the reader is referred to the web version of this article.)

HEK293T cells were also cultured on TCPS, PDMS, and PDMS with Cellbinder coating to investigate both biocompatibility and cell binding properties (Fig. 3). Cell spreading on PDMS was less than on TCPS and cells remained rounded with reduced visible cytoskeleton and nuclei closer together. When cultured on PDMS coated with Cellbinder, more focal adhesion points of the cytoskeleton were seen, indicating improved attachment and less stress.

**Fig. 4:**
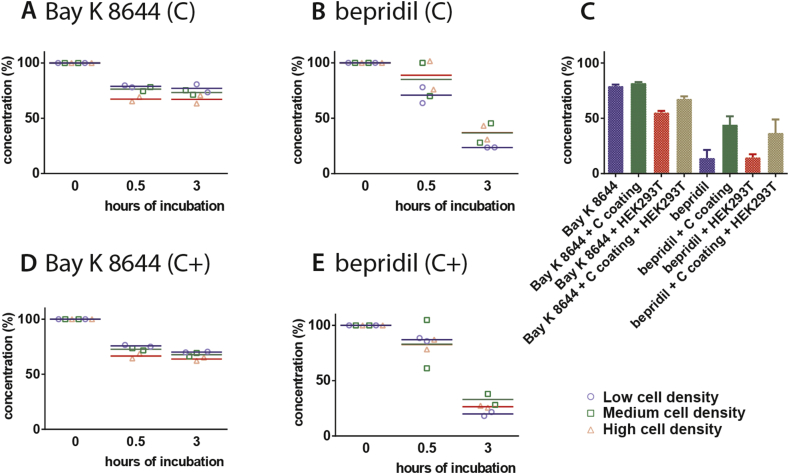
**Effect of cell density on coated PDMS**. A), B), D) and E) show plots of the free drug concentration of Bay K 8644 and bepridil after 0, 0.5 and 3 h of incubation at low, medium and high cell plating densities on PDMS coated with Cellbinder+ (C+) and Cellbinder (C). C) Summary of the effect of the final free drug concentration of Bay K 8644 and bepridil after 3 h of incubation on PDMS, Cellbinder coated PDMS, PDMS plus HEK293T cell culture and Cellbinder coated PDMS plus HEK293T cell culture. Averages from two independent experiments are shown.

Finally, different densities of HEK293T cells were cultured on Cellbinder and Cellbinder + -coated PDMS substrates and subsequently incubated with Bay K 8644 and bepridil to investigate the effect on free drug concentration (Fig. 4). For Bay K 8644, both coatings showed increased absorption at the highest cell density after 0.5 and 3 h, as also shown for uncoated PDMS plus HEK293T cell culture. For bepridil, there was a difference between the low and high cell density on the Cellbinder coating after 0.5 h, but this was not sustained at 3 h.

By analyzing the values found for free drug concentrations of Bay K 8644 and bepridil incubation after 3 h on different substrates with and without HEK293T cell culture (medium density) we compared the effect of the substrate conditions on the final free drug concentrations. Fig. 4C shows a decrease in Bay K 8644 concentration on PDMS with HEK293T culture, while there was no difference between PDMS, coated PDMS and coated PDMS with HEK293T culture. However, the loss of bepridil concentration on coated substrates was reduced compared to the uncoated substrates; there was no effect of the absence or presence of cell cultures.

## 5 Discussion

Cell culture devices made from PDMS are widely used in multiple formats but may bind molecules selectively from the medium, reducing their effective availability to cells. The study here showed that several cardio-active compounds differed in their absorption by TCPS-wells with PDMS base versus standard TCPS-wells. The presence of PDMS significantly affected the free concentration of compounds. Bepridil was most highly absorbed by TCPS with PDMS base after 3 h, followed by verapamil. Nifedipine and Bay K 8644 retained the highest free drug levels after the longest incubation time. No relationship was found between the reduction after incubation on TCPS with PDMS base and that on standard TCPS: for example, Bay K 8644 was reduced equally on both substrates whilst verapamil was reduced >50% on PDMS but not on TCPS. After 3 h of incubation, all compounds reached steady state (<10% concentration change) on PDMS, indicating saturation of the substrate. We estimated that the total reduction of bepridil concentration would be ±85% with a surface area of 0.3 cm^2^ and volume of 250 μL, indicating a free concentration of 150 nM instead of 1 μM after 3 h incubation. Typically, in a microfluidic chamber, the volume-to-surface ratio would be lower. For example, a PDMS microfluidic channel might normally have dimensions 50 × 5 mm length x width and 200 μm height, equivalent to 5.20 cm^2^ surface and 50 μL volume. Since the volume-to-surface ratio increases more than 86 times, the reduction in free compound concentration could be expected to increase similarly, highlighting the importance of this issue.

According to the log P threshold of 2.67 based on five compounds predicted by Wang et al. all compounds we examined should have shown high absorption but this was not the case. More importantly, we were unable to find a clear correlation between absorption and log P, suggesting that hydrophobicity is not the (single) parameter determining compound absorption by PDMS as proposed by Wang et al. Molecular weight of the compounds was also not correlated with the absorption by PDMS, nor did a combination of the log P value and molecular weight. We conclude that hydrophobicity and molecular weight are not the (only) parameters determining small molecule drug binding as previously suggested. Topological polar surface area indicated a linear relationship with absorption, but this should be confirmed by measurements with more compounds.

Ways to reduce compound absorption by PDMS specifically are of great interest. Two variants of a commercially available coating for PDMS were tested. These differed in chemical stability according to the manufacturer: Cellbinder+ is stable when exposed to air, whilst the regular variant of Cellbinder must be kept hydrated. The coatings are lipid-based and designed to mimic natural cell membrane interface. These types of materials are resistant to surface accumulation of proteins and can also act as barriers, preventing passage of molecules through the layer [22]. Both coatings had similar blocking properties with respect to the absorption by PDMS. They reduced the high absorption of bepridil by PDMS of >80% after 3 h to approximately 50%. Also, absorption of Bay K 8644 was reduced compared to uncoated surfaces, although only slightly.

HEK293T cells were used as a model to investigate the biocompatibility of the coating and also whether cells confounded absorption. HEK293T cells express endogenous calcium and sodium channels [27], [28], although these calcium channels are insensitive to the calcium channel specific Bay K 8644 [28]. The binding of bepridil was expected to be higher since it is non-selective compound and known to block both calcium and sodium channels [29]. However, after incubation of 3 h on PDMS plated with HEK293T cells, the free concentration of Bay K 8644 was highly reduced compared to incubation on PDMS only. The effect of compound absorption by cells is more pronounced than their potential to block compound absorption by the substrate, showing greater reduced free compound concentration at the high cell density compared to the low cell density. In contrast, the absorption of bepridil was not affected by the presence of cells. Since the absorption of bepridil by PDMS is very high, any reduction caused by the introduction of the HEK293T cells might be masked by the dominant substrate effect.

For PDMS coatings to be of practical use in cell culture assays they should be biocompatible and support cell attachment. Uncoated PDMS is biocompatible, but cells only attach following surface treatments such as the exposure to an oxygen plasma or ultra violet light [30], [31]. However, these treatments would destroy organic molecules like Cellbinder. An ideal coating would facilitate similar or better cell adherence than regular PDMS, without surface pre-treatment. Although most focal adhesion points were evident in HEK293T cells cultured on TCPS and the cytoskeleton showed greater cell spreading, Cellbinder-coated PDMS also supported adequate cell adherence, demonstrating its potential utility through both biocompatibility and cell adherence although adherence was lower than on TCPS.

Cellbinder-coated PDMS to which HEK293T cells were attached showed reduced compound absorption, both for Bay K 8644 and bepridil. The reduction in Bay K 8644 reached steady state within 0.5 h and the free concentration remained high for at least 3 h compared to uncoated PDMS with HEK293T cells. For both compounds, no differences were found between coated PDMS with and without cells, indicating that the coatings remained intact during cell culture and stable over longer periods of cell culture.

Overall, the study clearly showed the importance of determining the residual compound in the incubation fluid at the end of cellular dose response bioassays as part of dose response validation.

## Funding

Work in the Mummery lab is supported by the European Research Council [grant number ERCAdG 323182 STEMCARDIOVASC]. NC3Rs CRACK-IT [grant number 35911-259146] supported work in both the Mummery and Denning labs.

## Author disclosure statement

J. van Weerd, P. Jonkheijm and H.B.J. Karperien are co-founders of LipoCoat.
